# Supplementary material for: Unequal climate impacts on global values of natural capital
Source: Nature. 2023 Dec 18;625(7996):722–7. doi: 10.1038/s41586-023-06769-z (PMC10808060; doi:10.1038/s41586-023-06769-z)
Supplement: Supplementary file 1 — This file contains three supplementary tables that show: (1) the countries missing from the analysis; (2) the damage function coefficient for each country; and (3) the number of studies by type of ecosystem service in the VEGS database. [file 41586_2023_6769_MOESM1_ESM.docx]

**Supplementary Information for**

**Unequal Climate Impacts on Global Values of Natural Capital**

B. A. Bastien-Olvera*, M. N. Conte, X. Dong, T. Briceno, D. Batker, J. Emmerling, M. Tavoni, F. Granella, F. C. Moore

*Corresponding author email: [bbastien@ucsd.edu](mailto:bastien@ucdavis.edu)

Supplementary Table 1. Countries missing from the analysis due to data availability. If the country is not present in the wealth accounts, the algorithm was interrupted before attempting to extract the data from the vegetation models (shown as NA).

| **Country** | **Wealth Accounts** | **Vegetation Model** |
| --- | --- | --- |
| Bahrain | Available | Missing |
| Comoros | Available | Missing |
| Malta | Available | Missing |
| Mauritius | Available | Missing |
| Maldives | Available | Missing |
| Singapore | Available | Missing |
| Afghanistan | Missing | NA |
| Angola | Missing | NA |
| Brunei Darussalam | Missing | NA |
| Bahamas | Missing | NA |
| Bhutan | Missing | NA |
| Cuba | Missing | NA |
| Cyprus | Missing | NA |
| Algeria | Missing | NA |
| Eritrea | Missing | NA |
| Fiji | Missing | NA |
| Greenland | Missing | NA |
| Equatorial Guinea | Missing | NA |
| Guinea-Bissau | Missing | NA |
| Israel | Missing | NA |
| Dem. Rep. Korea | Missing | NA |
| Libya | Missing | NA |
| Montenegro | Missing | NA |
| Myanmar | Missing | NA |
| New Caledonia | Missing | NA |
| New Zealand | Missing | NA |
| Puerto Rico | Missing | NA |
| Serbia | Missing | NA |
| Sudan | Missing | NA |
| Somalia | Missing | NA |
| South Sudan | Missing | NA |
| Syria | Missing | NA |
| Timor-Leste | Missing | NA |
| Uzbekistan | Missing | NA |
| Vanuatu | Missing | NA |
| Somaliland | Missing | NA |
| Northern Cyprus | Missing | NA |

Supplementary Table 2. Natural capital damage coefficients.

| country | coef_nN | pval_nN | coef_mN | pval_mN |
| --- | --- | --- | --- | --- |
| ARE | -0.05494 | 0.556831 | -0.05631 | 0.567178 |
| ALB | -0.05388 | 2.62E-08 | -0.05946 | 4.44E-09 |
| ARM | 0.014738 | 0.093695 | 0.016348 | 0.027823 |
| ARG | -0.01298 | 0.038069 | -0.0114 | 0.068208 |
| AUT | -0.02459 | 0.000426 | -0.0054 | 0.471714 |
| AUS | 0.017532 | 0.324111 | 0.001613 | 0.922573 |
| AZE | -0.00962 | 0.507855 | 0.083819 | 5.56E-08 |
| BIH | -0.06763 | 1.61E-12 | -0.07165 | 3.48E-13 |
| BGD | 0.011142 | 0.257379 | 0.010832 | 0.293913 |
| BEL | -0.05734 | 6.88E-06 | -0.05152 | 2.25E-06 |
| BFA | -0.10439 | 2.91E-17 | -0.10297 | 2.24E-17 |
| BGR | -0.09435 | 1.88E-09 | -0.08225 | 1.19E-09 |
| BDI | 0.011908 | 0.390736 | 0.008165 | 0.548093 |
| BEN | -0.11335 | 2.56E-17 | -0.12128 | 4.45E-17 |
| BOL | -0.01054 | 0.196471 | -0.01715 | 0.022655 |
| BRA | -0.00934 | 0.383598 | -0.01755 | 0.001326 |
| BWA | 0.032101 | 0.130219 | 0.065838 | 0.01429 |
| BLR | 0.002813 | 0.692546 | 0.003986 | 0.544339 |
| BLZ | -0.08159 | 0.000203 | -0.07066 | 5.04E-05 |
| CAN | -0.02724 | 2.24E-08 | -0.02766 | 3.09E-07 |
| COD | 0.002061 | 0.552195 | 0.004058 | 0.243416 |
| CAF | -0.0018 | 0.655752 | -0.00538 | 0.134582 |
| COG | -0.00279 | 0.378145 | -0.0022 | 0.489099 |
| CHE | 0.005375 | 0.502631 | -0.00632 | 0.349159 |
| CIV | -0.034 | 4.13E-08 | -0.02644 | 3.70E-05 |
| CHL | 0.002755 | 0.286051 | 0.005511 | 0.038067 |
| CMR | 0.002224 | 0.426127 | 0.003862 | 0.171351 |
| CHN | 0.006124 | 0.10064 | 0.009048 | 0.04048 |
| COL | -0.0014 | 0.694102 | -0.00111 | 0.755274 |
| CRI | -0.04318 | 0.003807 | -0.04364 | 0.002797 |
| CZE | -0.04623 | 4.16E-07 | -0.03816 | 1.95E-05 |
| DEU | -0.05876 | 9.26E-12 | -0.05858 | 1.57E-12 |
| DJI | -0.13793 | 0.008566 | -0.13236 | 0.013278 |
| DNK | -0.02114 | 0.001262 | -0.01739 | 0.007902 |
| DOM | -0.0814 | 8.09E-06 | -0.07197 | 2.54E-06 |
| ECU | 0.010201 | 0.003309 | 0.010489 | 0.003206 |
| EST | 0.023642 | 7.86E-06 | 0.022493 | 2.20E-08 |
| EGY | -0.22933 | 1.86E-08 | -0.22592 | 2.05E-08 |
| ESP | -0.03983 | 5.26E-08 | -0.0323 | 1.23E-05 |
| ETH | -0.04804 | 0.003623 | -0.07484 | 3.18E-07 |
| FIN | 0.001786 | 0.907118 | -0.01982 | 0.000639 |
| FRA | -0.02862 | 5.36E-05 | -0.02878 | 1.17E-07 |
| GAB | -0.00626 | 0.004861 | -0.00608 | 0.000377 |
| GBR | -0.03832 | 1.51E-07 | -0.03765 | 5.32E-07 |
| GEO | 0.019699 | 4.02E-07 | 0.012765 | 1.06E-05 |
| GHA | -0.07052 | 1.85E-14 | -0.07236 | 4.97E-15 |
| GMB | -0.08137 | 0.000106 | -0.0879 | 1.65E-05 |
| GIN | -0.04688 | 1.10E-09 | -0.04855 | 1.49E-09 |
| GRC | -0.03286 | 0.011158 | -0.00824 | 0.401774 |
| GTM | -0.05259 | 0.000107 | -0.05353 | 3.20E-05 |
| GUY | -0.09354 | 8.69E-06 | -0.08291 | 2.88E-07 |
| HND | -0.06827 | 0.000373 | -0.07255 | 4.46E-06 |
| HRV | -0.07225 | 4.82E-12 | -0.06714 | 1.46E-11 |
| HTI | -0.07369 | 0.00011 | -0.07918 | 4.53E-07 |
| HUN | -0.08022 | 1.75E-06 | -0.0785 | 4.84E-06 |
| IDN | 3.30E-05 | 0.992621 | 0.000906 | 0.793756 |
| IRL | -0.07642 | 2.29E-13 | -0.07038 | 1.97E-11 |
| IND | -0.05848 | 2.20E-09 | -0.05438 | 1.29E-08 |
| IRQ | -0.09313 | 0.006831 | -0.09929 | 0.001326 |
| IRN | -0.01025 | 0.691726 | -0.00857 | 0.748056 |
| ISL | 0.174105 | 0.000191 | 0.169309 | 0.000483 |
| ITA | -0.02735 | 9.90E-05 | -0.03803 | 1.13E-05 |
| JAM | -0.02194 | 0.22034 | -0.02242 | 0.112101 |
| JOR | -0.06912 | 0.00863 | -0.06018 | 0.007467 |
| JPN | 0.008759 | 0.002135 | 0.008697 | 1.62E-05 |
| KEN | -0.11258 | 2.69E-14 | -0.10422 | 1.77E-12 |
| KGZ | -0.02628 | 0.011709 | -0.02279 | 0.062688 |
| KHM | 0.01019 | 0.235039 | 0.010882 | 0.212053 |
| KOR | -0.03547 | 1.39E-06 | -0.03402 | 2.92E-08 |
| KWT | -0.27504 | 0.000797 | -0.28673 | 9.40E-05 |
| KAZ | 0.007928 | 0.374704 | 0.01199 | 0.2168 |
| LAO | -0.01685 | 0.161667 | -0.01609 | 0.111723 |
| LBN | -0.01272 | 0.398159 | -0.00491 | 0.715666 |
| LKA | 0.019063 | 0.073065 | 0.001535 | 0.854467 |
| LBR | -0.01333 | 0.007105 | -0.01393 | 0.001679 |
| LSO | -0.15586 | 2.12E-15 | -0.07321 | 2.34E-05 |
| LTU | 0.022277 | 0.013288 | 0.021952 | 0.005742 |
| LUX | -0.08327 | 4.13E-08 | -0.09089 | 4.94E-10 |
| LVA | 0.01107 | 0.073032 | 0.009124 | 0.130414 |
| MAR | -0.08904 | 0.000106 | -0.08887 | 3.53E-05 |
| MDA | -0.05812 | 0.009821 | -0.05787 | 0.008824 |
| MDG | -0.03698 | 1.28E-13 | -0.02713 | 3.37E-11 |
| MKD | -0.05653 | 0.000519 | -0.04291 | 0.003067 |
| MLI | -0.06756 | 0.007071 | -0.06934 | 0.00719 |
| MNG | 0.00732 | 0.311373 | 0.009308 | 0.224026 |
| MRT | -0.05201 | 0.182173 | -0.05557 | 0.155615 |
| MWI | -0.05415 | 9.03E-16 | -0.07886 | 2.03E-17 |
| MEX | -0.04486 | 3.93E-06 | -0.05398 | 9.71E-09 |
| MYS | -0.0012 | 0.650175 | -0.00063 | 0.802187 |
| MOZ | -0.08725 | 1.43E-20 | -0.09323 | 2.39E-20 |
| NAM | -0.08827 | 8.70E-06 | -0.01596 | 0.494122 |
| NER | 0.052129 | 0.127522 | 0.06057 | 0.099071 |
| NGA | -0.03289 | 1.13E-16 | -0.03371 | 1.35E-16 |
| NIC | -0.08571 | 1.34E-05 | -0.08187 | 5.61E-07 |
| NLD | -0.04881 | 1.75E-05 | -0.05626 | 1.27E-05 |
| NOR | -0.083 | 4.06E-21 | -0.0781 | 8.13E-21 |
| NPL | -0.03187 | 0.002104 | -0.02893 | 0.004479 |
| OMN | 0.131787 | 0.057024 | 0.153273 | 0.035377 |
| PAN | -0.04666 | 9.27E-05 | -0.03855 | 0.000138 |
| PER | -0.00353 | 0.492718 | 0.001404 | 0.760154 |
| PNG | -0.00322 | 0.558616 | -0.00447 | 0.389957 |
| PHL | 0.00995 | 0.050872 | 0.010837 | 0.038488 |
| PAK | -0.01603 | 0.55159 | -0.00341 | 0.906017 |
| POL | -0.02021 | 0.00925 | -0.01894 | 0.019857 |
| PRT | -0.07958 | 8.26E-06 | -0.06042 | 1.38E-05 |
| PRY | -0.06232 | 4.71E-05 | -0.07278 | 2.09E-06 |
| QAT | -0.06583 | 0.423959 | -0.06811 | 0.441152 |
| ROU | -0.05679 | 9.44E-08 | -0.04133 | 3.36E-06 |
| RUS | -0.04805 | 9.20E-16 | -0.02925 | 4.48E-07 |
| RWA | 0.020293 | 0.148525 | 0.018446 | 0.190141 |
| SAU | -0.02811 | 0.488586 | -0.01824 | 0.664007 |
| SLB | -0.00894 | 0.258634 | -0.0107 | 0.149 |
| SWE | -0.01222 | 0.009035 | -0.03984 | 3.84E-12 |
| SVN | -0.09535 | 3.68E-09 | -0.09327 | 2.17E-10 |
| SVK | -0.02684 | 0.001087 | -0.0295 | 1.47E-05 |
| SLE | -0.01065 | 0.350346 | -0.01188 | 0.242255 |
| SEN | -0.14864 | 9.33E-15 | -0.14928 | 8.62E-16 |
| SUR | -0.08338 | 6.77E-05 | -0.07606 | 1.25E-05 |
| SLV | -0.14404 | 3.28E-07 | -0.15396 | 2.02E-07 |
| SWZ | -0.11182 | 1.73E-09 | -0.11983 | 4.21E-08 |
| TCD | -0.06404 | 0.000315 | -0.06655 | 0.000476 |
| TGO | -0.07108 | 7.02E-09 | -0.07542 | 1.42E-09 |
| THA | -0.02467 | 0.011866 | -0.02056 | 0.047107 |
| TJK | -0.00815 | 0.551562 | -0.00965 | 0.524086 |
| TUN | -0.09655 | 0.000885 | -0.10303 | 0.000115 |
| TUR | 0.022961 | 0.020356 | 0.037286 | 0.000144 |
| TTO | -0.11447 | 2.19E-05 | -0.09322 | 3.88E-08 |
| TZA | -0.08932 | 2.79E-16 | -0.1013 | 2.51E-18 |
| UKR | -0.00956 | 0.333329 | -0.01106 | 0.284024 |
| UGA | -0.0772 | 1.49E-08 | -0.0238 | 0.055772 |
| USA | 0.006435 | 0.110166 | 0.012295 | 0.002139 |
| URY | 0.004279 | 0.729868 | 0.003827 | 0.730932 |
| VNM | 0.003101 | 0.675846 | -0.00511 | 0.485546 |
| YEM | 0.19589 | 0.003681 | 0.206992 | 0.002973 |
| ZAF | -0.0866 | 3.89E-16 | -0.05774 | 8.71E-09 |
| ZMB | -0.09646 | 1.35E-21 | -0.10147 | 1.94E-22 |
| ZWE | -0.04039 | 0.026338 | -0.03421 | 0.084881 |

Supplementary Table 3. Number of observations in the VEGS database by Ecosystem Service Subcategory under three categories: Information (n=178), Provisioning (n=152), Regulating (n=474), Supporting (n=81).

| Aesthetic Information | 24 |
| --- | --- |
| Air Quality | 21 |
| Biological Control | 26 |
| Climate Stability | 99 |
| Cultural Value | 33 |
| Disaster Risk Reduction | 59 |
| Energy & Raw Materials | 116 |
| Habitat | 81 |
| Medicinal Resources | 3 |
| Ornamental Resources | 4 |
| Pollination & Seed Dispersal | 30 |
| Recreation & Tourism | 82 |
| Science & Education | 39 |
| Soil Formation | 21 |
| Soil Quality | 32 |
| Soil Retention | 32 |
| Water Capture, Conveyance, & Supply | 67 |
| Water Quality | 87 |
| Water Storage | 29 |
